# Dosimetric evaluation of surface‐guided tattoo‐free approach for right sided whole‐breast irradiation

**DOI:** 10.1002/acm2.70294

**Published:** 2025-10-09

**Authors:** Xinxin Zhang, Chengzhu Zhang, Swati Mamidanna, Yin Zhang, Xiao Wang, Ning J Yue, Shicha Kumar, Maria J Kowzun, Lindsay Potdevin, Mridula George, Lara Hathout, Bruce Haffty, Nisha Ohri

**Affiliations:** ^1^ Department of Radiation Oncology Rutgers Cancer Institute of New Jersey, Rutgers Robert Wood Johnson Medical School New Brunswick New Jersey USA; ^2^ Department of Surgical Oncology Rutgers Cancer Institute of New Jersey, Rutgers Robert Wood Johnson Medical School New Brunswick New Jersey USA; ^3^ Department of Medical Oncology Rutgers Cancer Institute of New Jersey, Rutgers Robert Wood Johnson Medical School New Brunswick New Jersey USA

**Keywords:** dosimetric evaluation, surface‐guided radiation therapy, tattoo‐less

## Abstract

**Background and purpose:**

In preparation for adjuvant breast radiation therapy (RT), permanent skin tattoo marks are often placed on the patient's skin to assist patient positioning on the treatment couch. However, those marks are often undesirable, in particular, for breast cancer patients due to various cosmetic and psychological concerns and other drawbacks. With surface‐guided radiation therapy (SGRT) being readily available, it becomes possible to adopt a “tattoo‐free” approach for patient setup. This study evaluates the efficacy and dosimetric implications of a tattoo‐free setup technique.

**Methods:**

Thirty right‐sided breast cancer patients were included in this retrospective study. All patients received an initial course of whole breast treatment of 42.56 Gy in 16 fractions using a tangential 3D conformal technique, followed by a 10 Gy boost to the lumpectomy site in four fractions. SGRT was used in daily setup to reproduce patient's positioning between the simulation and treatment. The patient's breast surface was aligned with the corresponding reference breast surface generated from the planning computed tomography (CT) images via AlignRT system. To evaluate the feasibility and accuracy of the new tattoo‐free approach, at the beginning of the program and for this group of patients, daily orthogonal kV imaging pair was performed to confirm the treatment positioning by verifying the bony landmarks, and the translational couch shifts were recorded for every fraction. Those shifts were applied to the isocenter positions of the original corresponding clinical treatment plans, and dose distributions were re‐computed. The dosimetric evaluation between the two setup methods, that is, the breast surface alignment versus the bony landmark alignment, were then assessed on the original clinical plan. New plan sums were obtained from the 16 fractions, where each one was recalculated based on the new isocenter positions determined with the kV imaging shifts. Boost fractions were excluded in this study, as setup was verified using surgical clips as the matching reference.

**Results:**

Translational shifts for the 30 patients (*N* = 480) were reviewed. The mean absolute shift resulted from the orthogonal kV imaging‐based setup following the SGRT setup, in lateral, vertical and longitudinal directions, were 0.20 cm (ranged from 0 to 1.98 cm with 95% confidence interval (CI) of 0.18–0.22 cm), 0.23 cm (ranged from 0–1.25 cm with 95% CI of 0.21–0.25 cm), and 0.22 cm (ranged from 0 to 1.35 cm with 95% CI of 0.19–0.24 cm), respectively. The average change in V95% coverage over the treatment course of the 30 patients was 0.99%. No significant differences in V20Gy of lung (%) and mean heart dose were observed between the original and the corresponding shifted plans.

**Conclusion:**

A SGRT based tattoo‐free setup approach was clinically evaluated and compared to a kV orthogonal imaging‐based approach for whole breast RT treatment. It was found that the tattoo‐free setup approach is acceptable in treatment setup accuracy and dosimetric coverage. Caution needs to be paid to patient movement during setup and treatment to ensure the safety and efficacy of the approach.

## 1 INTRODUCTION

Permanent skin tattoos have been used as a standard approach to position patients in treatment rooms in preparation for receiving radiation therapy (RT). However, studies have shown many drawbacks of using tattoo marks, in particular, for breast cancer patients. Although these marks are small, they may still have a negative impact on a patient's self‐confidence and be a concern when choosing appropriate clothing and swimwear.^1^ In fact, these marks may be a constant reminder of their cancer diagnosis, even after the radiation treatments have been completed.^2^ Cultural or religious beliefs, pain tolerance level, mobility of skin on elderly or large patients are all common reasons why patients may be resistant to receiving tattoo marks.^1^ With the availability of surface‐guided radiation therapy (SGRT) technique,^3^ a tattoo‐free approach for patient positioning has become feasible and warrants investigation for clinical implementation.

In whole‐breast RT, it is now a common practice that patients receive 42.56 Gray (Gy) in a hypofractionated schedule of 16 fractions followed by a seroma/scar boost of 10 Gy when indicated,^4^ or 50 Gy in a conventional schedule of 25 fractions.^5^, ^6^ Traditionally, the patient setup is performed by aligning to the tattoo marks. Subsequent kV or MV imaging can be an option to further ensure patient position. In the SGRT based tattoo‐free approach, the breast surface is used as the primary reference for daily patient setup. A large body of literature reported the feasibility of a SGRT based tattoo‐free approach in lieu of tattoo marks for initial patient setup with sufficient accuracy and time efficiency.^7^, ^8^, ^9^, ^10^, ^11^, ^12^, ^13^ Those studies focused on the comparisons of setup accuracy between the tattoo and tattoo‐free approaches by reporting mostly the translational positioning shifts derived from the kV or MV imaging registration, however, little was reported on the dosimetric comparisons between the two setup methods. In this study, an SGRT based tattoo‐free approach was first used in daily setup for treatment. This was followed by an orthogonal kV imaging pair where patient anatomic structures were used as the primary reference for online matching, resulting in some possible translational shifts that could cause dosimetric differences between the two positions. The current study was undertaken to investigate dosimetric differences. In the study, it was assumed that kV imaging determined position corresponded to that of the original clinical plan.

## 2 METHODS

### 2.1 Study population

Thirty right‐sided breast cancer patients who received adjuvant whole breast RT were included in this study. All patients received an initial course of whole breast dose of 42.56 Gy in 16 daily fractions using a tangential 3D conformal planning technique. The surface‐guided setup and orthogonal kV imaging pair setup were performed consecutively for each daily fraction. The boost treatment was not included in this study, as the onboard imaging is usually matched to their surgical clips or postoperative seroma instead of anatomic structures. In addition, patients who exhibited significant breast anatomy changes over the course of treatment were not included, as SGRT was not used for setup and the evaluation of setup uncertainty induced by such changes is out of the scope of this study. Table 1 shows the demographic data for the 30 patients.

**TABLE 1:** Summarization of the demographic data.

Number of patients	30
Age (mean, range, years)	60 (36, 79)
Breast volume (mean, range, cc)	853.5 (263.3, 1733.2)

### 2.2 Treatment setup via surface‐guided imaging

**FIGURE 1:**
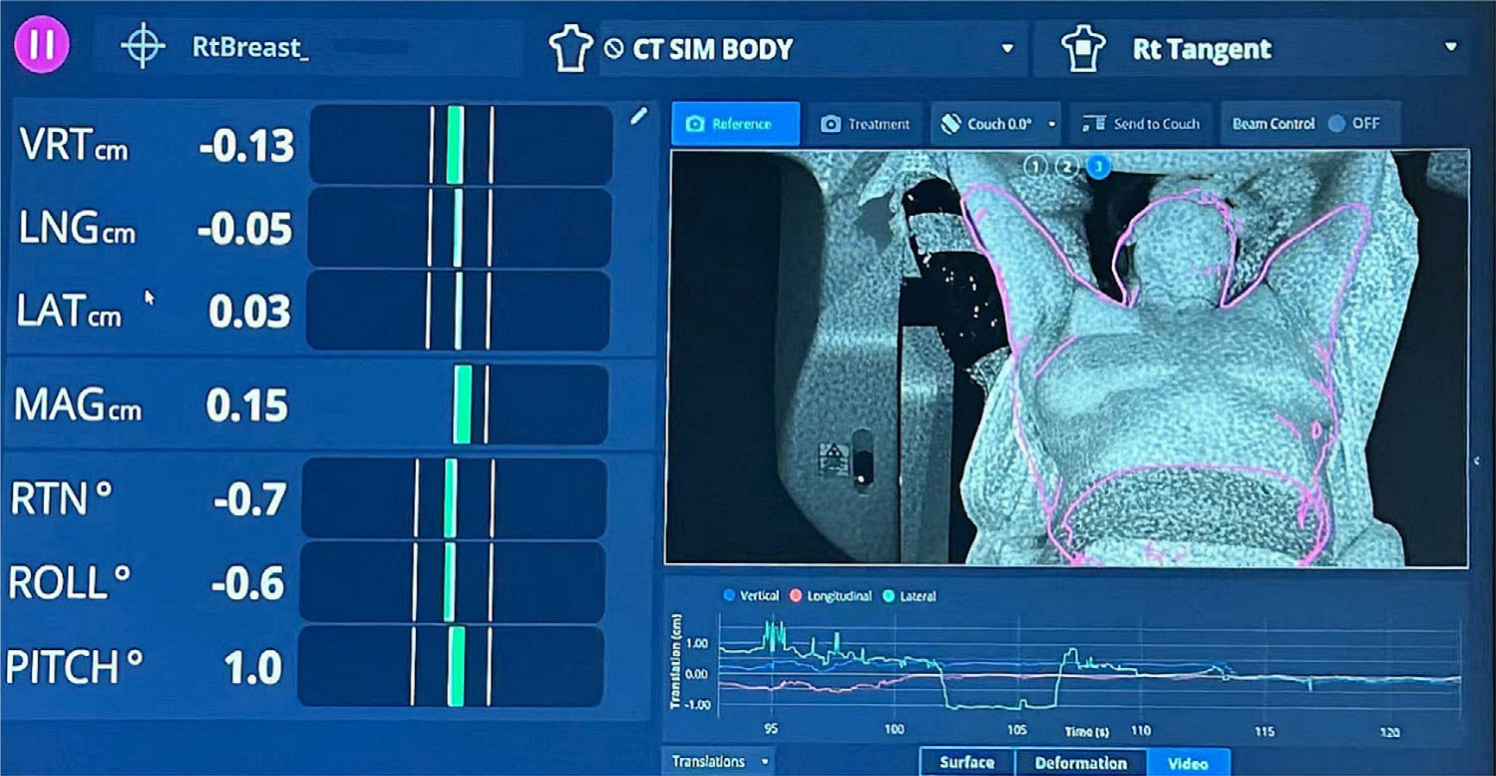
The software interface of AlignRT system for a right‐side patient in the treatment room. The left panel shows the 6‐degree setup tolerance comparing to the reference ROI drawn on the planning CT. Green error bars shown on the left column indicate the setup is within tolerance. The right panel shows the postural setup.

The commercially available AlignRT system (Vision RT, London, UK) was used for SGRT in our department. The system has three camera pods mounted in the treatment room to capture patient's position on the treatment couch and generate a 3D surface model of the region of interest (ROI) of patient body. During the setup, this modeled 3D surface is registered with a reference surface of patient body, and the determined differences in patient positioning are used as the guidance for patient treatment positioning adjustment. The reference surface is generated from the simulation computed tomography (CT) images of the corresponding treatment plan and exported to the AlignRT system. A typical example of the modeled 3D patient surface using the postural video feature of the AlignRT system is presented in Figure 1.

At the treatment simulation, patients were immobilized on an indexed breast wing board with customized upper alpha cradle, with both arms up and chin turned to the contralateral side of the treated breast when indicated. Free‐breathing CT scan with 2.5 mm slice thickness was acquired with an GE Lightspeed RT 16 scanner (GE, Wakesha, WI). The clinical treatment plans were generated using the Eclipse treatment planning system (TPS) (Varian Medical Systems, a Siemens Health Company, Palo Alto, CA). By correlating the coordinate systems among the CT simulation images, the treatment couch and the treatment machine isocenter, the initial absolute treatment couch position was estimated from the clinical treatment plan for each individual patient and was made available as a starting point for therapists to set up a patient on the treatment machine. Following the initial couch position, the AlignRT system was used to fine tune the patient's position by matching the acquired breast surface to the corresponding reference surface. The default tolerances in AlignRT were 3 mm in the translational directions and 3 degrees in the rotational directions. Our clinical guidance for the rotational directions is 1 degree. Any translational and rotational offset were corrected to the best extent by manually positioning the patient to meet the tolerance requirement. Clinical decisions would be made if rotational offset exceeded 1 degree. The AlignRT system also provides a live video stream of a patient in treatment room and gives an outline of the reference surface in multi‐angle views to make the position alignment faster and ensure the correct patient position. This live video feature was also used during the setup in addition to the static surface view.

### 2.3 Setup verification via kV/MV imaging

After the surface guided treatment setup, orthogonal kV imaging pairs were acquired to verify the patient positioning. Anatomic structures, typically bony anatomy such as chest wall, were matched in the image registration between the kV images and the digitally reconstructed radiograph (DRR) created from the simulation CT images. Although no significant discrepancies in rotational directions were observed in the patients investigated in this work, the rotational shifts, if more than 1 degree, were corrected before treatment by manually moving the patients on the treatment couch. Observed translational shifts were applied to the treatment couch and recorded.

In addition to the kV images, a double‐exposure MV portal imaging pair (one with the treatment field opening and the other with larger field size to include adjacent patient anatomy of interest) was acquired on the first day of treatment and subsequently on a weekly basis to ensure correct patient's positioning, as well as to capture any significant anatomic changes. In cases where large shifts were observed during the online kV image matching, or the breast surface alignment exceeded the preset tolerance even after applying the kV imaging‐based shifts, MV portal images were acquired for additional positioning verification, and a new surface reference was captured in the AlignRT system to monitor intra‐fractional motion during treatment. If a new surface reference was captured for more than two consecutive treatments, the captured reference could be used as setup surface for the remaining treatments.

### 2.4 Dosimetric evaluations between the treatment setups via SGRT and kV imaging

To assess the dosimetric differences between the treatment setups via SGRT based breast surface and kV imaging based anatomic landmarks, a “virtual delivery” was performed in the Eclipse TPS. The dose distribution of the original clinical treatment plan was used to represent that of the kV imaging‐based setup method. At the same time, the translational shifts determined from the orthogonal kV imaging after the SGRT tattoo‐free setup represented the alignment differences resulted from using breast surface and kV imaging anatomy matching. Those translational shifts were further applied to the isocenter position of the original clinical plan to re‐compute the dose distribution of the SGRT tattoo‐free setup. The re‐computation was performed with all the identical plan parameters of the original plan, such as the field‐in‐field MLC design, the monitor unit number (MU) of each field, gantry angle, collimator angle, energy, and so forth. except for the isocenter location in patient anatomy. For each patient, a plan sum over all the 16 re‐computed fractions was conducted. The dosimetric impact from the alignment difference, that is, breast surface versus anatomic landmarks, was assessed by comparing the dose distributions of the clinical plan and the re‐computed plan and by evaluating how the re‐computed plans met our institutional breast cancer protocol.

Table 2 presents the dosimetric constraints employed in our institution for treating whole breast alone without regional nodal radiation in the hypo‐fractionated scheme. The breast structure evaluated in both clinical and re‐computed plans as the treatment target was generated using the AutoContour (RADformation, New York, NY) and reviewed by the radiation oncologist team.

**TABLE 2:** Dose constraints per CINJ breast cancer protocol (Hypo fractionated without nodes) for both breast and normal tissues with prescription of 42.56 Gy in 16 sequential fractions.

Target Volume	Goal	Volume	Dose
Breast	Ideal	≥ 95% of the breast PTV Eval receives	≥ 95% of prescribed whole breast dose
	Acceptable	≥ 90% of the breast PTV Eval receives	≥ 95% of prescribed whole breast dose
	Ideal	≤ 200 cc of the breast PTV Eval receives	≥ 105% of the prescribed whole breast dose
Breast		≤ 2 cc of the breast PTV Eval receives	≥ 107% of the prescribed whole breast dose
	Acceptable	≤ 200cc of the breast PTV Eval receives	≥ 107% of the prescribed whole breast dose

Normal tissue	Goal	Volume	Dose
Heart	Ideal	Mean dose	≤ 2 Gy
	Acceptable	Mean dose	≤ 4 Gy
Ipsilateral lung	Ideal	≤ 15% of the ipsilateral lung receives	≥ 16 Gy
	Acceptable	≤ 20% of the ipsilateral lung receives	≥ 16 Gy

### 2.5 Statistical analysis

**FIGURE 2:**
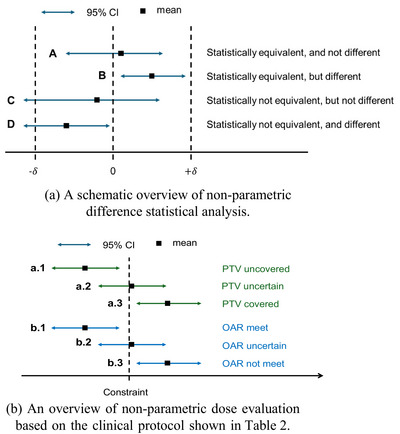
(a) A schematic overview of non‐parametric difference statistical analysis. (b) An overview of non‐parametric dose evaluation based on the clinical protocol shown in Table 2.

Shapiro‐wilk normality test was first performed on the parameter metrics‐of‐interest to evaluate their conformity to the normal distribution.^14^ Following the normality test result, non‐parametric analysis was performed using the bootstrapping method to quantify 95% confidence interval (CI) for each of the parameters of interest.^15^ One thousand repeated bootstrapping were performed and 30 samples (i.e., the size of our dataset) were drawn each time with replacement. The confidence interval was determined from the statistical mean value of 1000 repetitions. This non‐parametric approach allowed us to infer the variability of our data without making assumptions about the underlying data distribution. To establish an equivalency margin (δ) between the two datasets, the bootstrapping (still 1000 repeated retrieval of samples) based on two one‐sided equivalency test (TOST) was performed to establish the smallest equivalency margin that yields 95% of confidence.^16^ The null hypothesis was that the difference between the two comparison methods was larger than the defined margin δ. As shown in the overview of non‐parametric difference statistical analysis (Figure 2a), the statistical equivalence and difference can be determined from where the 95% CI resides. Non‐parametric dose evaluation was performed for target coverage and OARs, which compliances with our institutional clinical constraints were determined upon the calculations of the 95% CI, as shown in Figure 2b.

## 3 RESULTS

### 3.1 The relative translational shifts determined based on kV imaging

**FIGURE 3:**
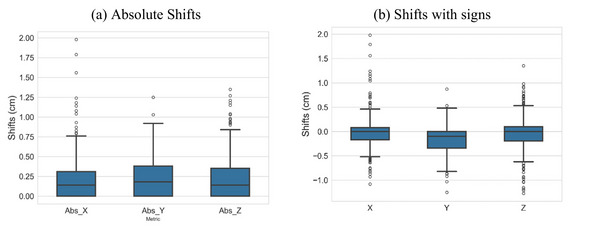
(a) The box‐whisker plot of absolute translational shifts from kV imaging; (b) The actual transitional shifts with signs. *X*, *Y*, and *Z* represent lateral, vertical, and longitudinal directions, respectively.

Table 3 shows the mean kV imaging‐based couch shifts from the initial SGRT based setup for all the 30 patients (*N* = 480). It was noted that the mean values of the shifts in all directions were almost zero, as demonstrated in Table 3. Absolute values were mostly the same in all directions. The reported shifts indicated the systematic errors were very small. Figure 3 exhibits the box‐whisker plot of all the kV imaging based relative translational shift results.

**TABLE 3:** Translational couch shifts according to kV imaging after the initial SGRT alignment.

Direction		Mean	95% CI	Range
Lateral (cm)	with signs	0.01	(−0.04, 0.02)	[−1.08, 1.98]
	absolute	0.20	(0.18, 0.22)	[0.00, 1.98]
Vertical (cm)	with signs	−0.15	(−0.18, −0.13)	[−1.25, 0.87]
	absolute	0.23	(0.21, 0.25)	[0.00, 1.25]
Longitudinal (cm)	with signs	−0.03	(−0.06, 0.01)	[−1.27, 1.35]
	absolute	0.22	(0.19, 0.24)	[0.00, 1.35]

### 3.2 The dosimetric evaluation results

**FIGURE 4:**
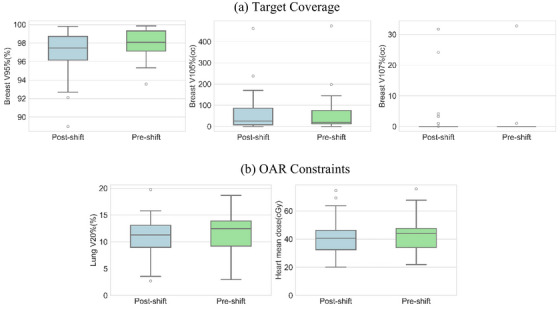
The dosimetric evaluation of (a) the target coverage and (b) OAR constraints. The green box plot shows the result in the original plan while the results of the plans with shifted isocenter are shown in blue box plot. Outliers were mainly displayed in V_107%_ (cc) because the majority of data are 0 cc.

Figure 4 and Table 4 present the dosimetric comparisons between the two treatment setup groups, that is, the kV imaging based and SGRT based treatment setups with detailed dosimetric metrics analysis. The Shapiro‐Wilk normality test showed that all dosimetric metrics deviated from the normal distribution with p values < 0.05 except for V_20Gy_ lungs (%) and the mean heart dose (cGy). It should be noted that the dosimetry metrics corresponding to the shifted isocenter locations (i.e., SGRT tattoo‐free treatment setup) all met the planning goals specified in Table 2 with 95% confidence. The target volume coverage metric V_95%_ (%) was found to be more than 95% with 95% confidence, that is, the lower bound of 95% CI is larger than 95%.

**TABLE 4:** Dosimetric evaluation on the recalculated plan with shifted isocenter and the original clinical plan for each dose constraint.

Metrics	Constraints (ideal)		Mean	95% CI	Meet with 95% confidence?
Breast V_95%_ (%)	> 95	shifted	96.89	(95.93, 97.68)	Yes
		original	97.87	(97.27, 98.44)	Yes
Lungs V_20Gy_ (%)	< 15	shifted	10.94	(9.63, 12.33)	Yes
		original	11.73	(10.32, 13.00)	Yes
Mean Heart (cGy)	< 200	shifted	41.27	(36.90, 45.95)	Yes
		original	42.90	(38.75, 47.46)	Yes
Breast V_105%_ (cc)	< 200	shifted	65.36	(34.45, 104.40)	Yes
		original	58.95	(32.84, 97.34)	Yes
Breast V_107%_(cc)	< 2	shifted	2.18	(0.18, 5.07)	Yes
		original	1.13	(0.00, 3.35)	Yes

The mean differences of each dosimetric constraint are reported in Table 5. The average variation in V_95%_ (%) of the target volume was within 1%, ranged from −6.67% to 3.27% with 95% CI of −1.63% to −0.48%. No significant differences were observed in V_20Gy_ of lung (%) and the mean heart dose (cGy) between the clinical and the corresponding shifted plans. This was indicated by the small equivalency margins established for V_95%_ (%), V_20Gy_ lung (%) and mean heart dose (cGy). The statistical test proved that the null hypothesis of the methods results in the difference larger than the defined margin was rejected with 95% confidence. A slight but not clinically significant increase was observed for V_105%_ (cc) and V_107%_ (cc).

**TABLE 5:** Dosimetric difference between the recalculated plan with shifted isocenter and the original clinical plan for each dose constraint.

Metrics	Mean	95% CI	Range	Equivalency margin δ
Breast V_95%_ (%)	−0.99	(−1.63, −0.48)	[−6.68, 3.27]	1.65
Lungs V_20Gy_ (%)	−0.79	(−1.16, −0.43)	[−3.73, 1.11]	1.20
Mean Heart (cGy)	−1.63	(−2.34, −0.96)	[−7.00, 1.60]	2.40
Breast V_105%_ (cc)	6.41	(−4.30, 21.34)	[−40.07, 35.59]	22.00
Breast V_107%_ (cc)	1.05	(0.04, 2.65)	[−1.07, 23.12]	3.00

## 4 DISCUSSION

In this study, the dosimetric differences between the SGRT based treatment setup using breast surface as the reference and the kV imaging‐based setup using anatomic landmarks for alignment were investigated for the right‐sided breast cancer patients who underwent hypo‐fractionated whole breast radiation treatment. No statistically significant setup bias (systematic error) was observed between the two treatment setup approaches.

Although the breast surface should be in its simulated position using the AlignRT system with the preset small alignment tolerance (3 mm), relatively large translational shifts were needed to match the kV imaging after the SGRT setup for some treatment fractions. The intrinsic discrepancy caused by the different surrogates for alignment (breast surface vs. bony landmarks) between the SGRT‐based setup and kV imaging‐based will result in consistent shifts after kV imaging acquisition, especially when significant anatomical change occurs.^17^ However, the large shifts (> 1 cm) observed in this study were not replicable among all treatment fractions for a patient and no such shifts were observed at the following fractions. The average shift throughout the treatment course for a patient was within sub‐centimeter. Therefore, these inconsistent and occasional large shifts were likely caused by patient movement after the SGRT setup and before the kV image acquisition. This observation indicates that patient movement needs to be carefully monitored at all times after the initial setup to the end of treatment, and additional imaging verification should be performed if sudden patient movement is observed. In addition, onboard imaging acquisition should be maintained at a minimum frequency of once per week for patient's position and anatomy verification throughout the treatment course. It also important to note that in the current study, patient setups were all under free‐breathing conditions. The observed free breathing induced setup uncertainty was generally small, and the SGRT tattoo‐free setup was relatively smooth. However, if a patient were to be positioned under the deep breathing condition, relatively large breast surface movement can happen more frequently, leading to challenges in surface alignment.

There were no significant dosimetric differences observed between the two treatment setup approaches. The primary intent of this study was to evaluate the dosimetric differences between the treatment setup methods, thus, TOST statistical test and analysis was used to establish the statistical equivalency margin. This test would allow a better understanding of the statistical differences of two datasets. As shown in Table 5, statistical differences between the two setup methods were indeed observed in dose constraints such as V_95%_ (%), V_20Gy_ lung (%), the mean heart dose (cGy). However, as shown in the last column of Table 5, the equivalency margin was found to be too small to have any meaningful impact.

The largest loss of coverage over the entire treatment course was found to be 6.67% for one patient whose target coverage V_95%_ (%) decreased from 95.65% to 88.98%. In this case, MV images were performed on several fractions of which large kV imaging shifts were observed, and additional shifts were applied again after the MV imaging, in addition to the kV imaging shifts. That was mainly caused by unusual patient movement after the breast surface alignment but before the kV imaging, and possibly even between the kV and MV imaging, too. In addition, a slight breast anatomical change was observed in MV images which could also add uncertainties to the shifts, resulting in under‐coverage of the target. This was the only patient whose V_95%_ (%) of the target decreased to an undesirable level (less than 90%), as this patient tended to move on treatment table, highlighting the importance of constant patient monitoring after initial setup and throughout the treatment.

Even though no significant breast anatomical changes were observed in all the enrolled patients, the evaluation of the complete dosimetric differences caused by the anatomic changes would be challenging with the current study design as the detailed soft tissue information was generally missed on the kV orthogonal 2D images. Potential future work incorporating onboard CBCT imaging can provide more detailed patient anatomical information to better assess the differences between the breast surface alignment and anatomic landmark alignment. In addition, our study indicates that intra‐fractional breast motion induced by patient's respiration and other spontaneous body movements should be constantly monitored with the SRGT technology and should be employed with a pre‐defined radiation beam gating tolerance to prevent the mistreatment caused by patient's large incidental intra‐fractional movement.

## 5 CONCLUSION

A SGRT based tattoo‐free setup approach was clinically evaluated and compared to a kV orthogonal imaging‐based approach for right‐sided whole breast RT treatment. It was found that the tattoo‐free setup approach is acceptable in treatment setup accuracy and dosimetric coverage. Caution needs to be paid to patient movement during setup and treatment, and possible intrinsic deviation between the breast surface and bony landmarks for alignment to ensure the safety and efficacy of the approach. Onboard imaging verification is essential in tattoo‐free setup, in addition to constant monitoring of patient movement during treatment. The frequency of imaging can be determined by individual cases, but should be maintained at a minimum frequency of once per week.

## AUTHOR CONTRIBUTIONS

Chengzhu Zhang, Yin Zhang, Xiao Wang, Ning J Yue, Nisha Ohri, and Xinxin Zhang conceived of the presented idea. Chengzhu Zhang and Xinxin Zhang led the computation, analysis, and writing of the manuscript. All authors reviewed and approved the final manuscript.

## CONFLICT OF INTEREST STATEMENT

The authors declare no conflicts of interest.
